# Development and internal validation of a prediction model for early identification of sepsis-associated acute kidney injury based on admission serum biomarkers: a retrospective cohort study

**DOI:** 10.3389/fmed.2026.1820595

**Published:** 2026-06-22

**Authors:** Chi Wang, Rui Ye, Xingxin Gong, Meng Tang, Fei Ding, Yi Xie, Yanxi Sheng, Xin Nie, Yong He

**Affiliations:** 1 Department of Laboratory Medicine, West China Hospital, Sichuan University, Chengdu, Sichuan, China; 2 Sichuan Clinical Research Center for Laboratory Medicine, Chengdu, Sichuan, China; 3 Clinical Laboratory Medicine Research Center, West China Hospital, Chengdu, Sichuan, China

**Keywords:** acute kidney injury, prediction model, risk stratification, sepsis, serum biomarkers

## Abstract

**Objective:**

Sepsis-associated acute kidney injury (SA-AKI) is a critical complication that substantially increases intensive care unit mortality. Early identification is paramount for timely intervention. This study aimed to develop and internally validate a prediction model relying exclusively on the first serum laboratory indicators after hospital admission to predict SA-AKI risk at the earliest available laboratory assessment.

**Methods:**

Clinical data of 1,573 sepsis patients admitted to West China Hospital of Sichuan University (January 2024–December 2025) were retrospectively analyzed. Patients were divided into SA-AKI and non-SA-AKI groups per 2012 KDIGO criteria, and randomly split into training (70%, *n* = 1,102) and validation (30%, *n* = 471) cohorts. LASSO regression screened potential predictors exclusively from first serum laboratory indicators after admission, and multivariate logistic regression constructed the model. A nomogram was constructed to facilitate individualized risk estimation. Subgroup analyses were performed across age and sex strata. Model performance was evaluated via ROC curves, calibration curves, and decision curve analysis (DCA).

**Results:**

LASSO regression selected 10 serum indicators, and multivariate logistic regression confirmed 8 independent risk factors: myoglobin (MYO), alanine aminotransferase (ALT), phosphorus (PO_4_), sodium (Na), potassium (K), carbon dioxide combining power (CO_2_-CP), platelet (PLT), and neutrophil (NEUT) (all *P* < 0.05). The model exhibited favorable discrimination (training cohort *AUC* = 0.839 [95% *CI*: 0.814–0.864]; validation cohort *AUC* = 0.832 [95% *CI*: 0.791–0.874]), acceptable graphical calibration, and significant clinical utility across a wide threshold probability range. Subgroup analyses showed stable discriminative performance across age and sex strata (all AUCs > 0.81).

**Conclusions:**

This study developed and internally validated a promising predictive model for estimating SA-AKI risk in sepsis patients using solely first routine serum laboratory indicators after admission. A nomogram is provided for individualized bedside risk estimation. This tool may support early risk stratification of high-risk individuals. External validation in multi-center, diverse cohorts is warranted before broader clinical implementation.

## 1 Introduction

Sepsis—life-threatening organ dysfunction resulting from a dysregulated host response to infection—remains a leading cause of morbidity and mortality in critically ill patients (1). The kidney is particularly susceptible to sepsis-related injury, with sepsis-associated acute kidney injury (SA-AKI) developing in a substantial proportion of hospitalized patients (2). SA-AKI is closely linked to multiple organ dysfunction, prolonged hospitalization, increased need for renal replacement therapy, and excess short- and long-term mortality, underscoring the urgent need for earlier recognition and risk stratification (3).

Despite its clinical importance, timely identification of SA-AKI remains challenging. Contemporary diagnosis largely follows the 2012 KDIGO criteria, which rely on changes in serum creatinine (Scr) and urine output (2). However, these markers are imperfect surrogates of real-time kidney injury. Scr may rise late relative to tubular damage and is strongly influenced by non-renal factors common in sepsis, including reduced creatinine generation (e.g., muscle hypoperfusion and catabolism), hemodilution from aggressive fluid resuscitation, and uncertainty in baseline Scr (4). Urine output, while often available, is non-specific and readily confounded by volume status, vasopressor use, diuretics, and hemodynamic instability (5). In the specific context of sepsis, these limitations are compounded—volume resuscitation further dilutes Scr, and sepsis-induced muscle wasting reduces creatinine production, rendering Scr an even less reliable marker of early renal injury. Consequently, KDIGO-based diagnosis may detect injury after a potentially preventable window has passed, limiting opportunities for early nephroprotective interventions.

Novel biomarkers [e.g., cystatin C, kidney injury molecule-1, and neutrophil gelatinase-associated lipocalin (NGAL)] have shown promise for earlier detection, yet single-marker strategies have not been adopted widely due to pathobiological heterogeneity, variable assay availability, and inconsistent performance across settings (6). In parallel, data-intensive machine-learning models can achieve good discrimination but often require numerous longitudinal variables (including urine output trajectories and baseline renal parameters) that may not be readily accessible or reliable at the time of the first post-admission laboratory assessment (7). Therefore, there remains a practical need for an interpretable prediction tool that relies solely on routinely obtained initial serum tests to identify patients at high risk for SA-AKI at the time of the first available laboratory data.

In this study, we developed and internally validated a prediction model for SA-AKI using only objective first serum biomarkers after hospital admission in patients with sepsis, aiming to provide a simple and scalable approach for early bedside risk stratification without incorporating clinical indicators or longitudinal data.

## 2 Methods

**Figure 1:**
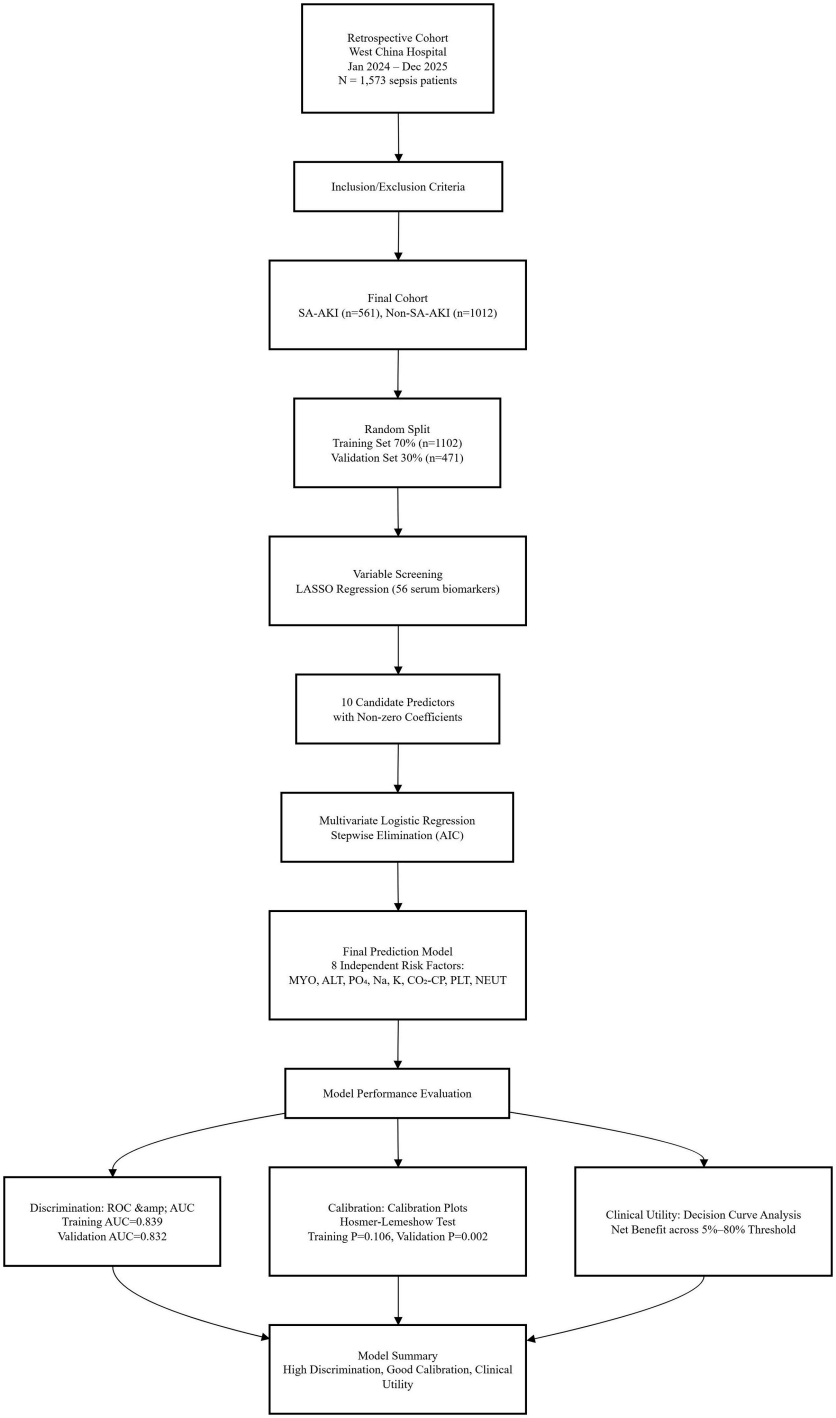
Flowchart of the development and internal validation process of the prediction model for sepsis-associated acute kidney injury (SA-AKI) based on admission serum biomarkers. This retrospective single-center cohort study enrolled 1,573 patients with sepsis admitted to West China Hospital between January 2024 and December 2025. After applying strict inclusion and exclusion criteria, 561 patients with SA-AKI (defined by KDIGO 2012 criteria) and 1,012 non-SA-AKI controls were included. The cohort was randomly split into a training set (70%, *n* = 1,102) and a validation set (30%, *n* = 471). In the training set, least absolute shrinkage and selection operator (LASSO) regression was applied to screen 56 serum biomarkers measured at admission, yielding 10 candidate predictors with non-zero coefficients. These were further refined using multivariate logistic regression with stepwise elimination, resulting in a final model comprising 8 independent risk factors: myoglobin (MYO), alanine aminotransferase (ALT), phosphorus (PO_4_), sodium (Na), potassium (K), carbon dioxide combining power (CO_2_-CP), platelet count (PLT), and neutrophil count (NEUT). Model performance was evaluated in both cohorts using receiver operating characteristic (ROC) curves (area under the curve, AUC), calibration plots with the Hosmer–Lemeshow test, and decision curve analysis (DCA). The model demonstrated excellent discrimination (training *AUC* = 0.839, 95% *CI* 0.814–0.864; validation *AUC* = 0.832, 95% *CI* 0.791–0.874), satisfactory calibration, and favorable clinical utility across a wide range of threshold probabilities (5%−80%).

Figure 1 outlines the stepwise process of study design, patient selection, model development, and internal validation.

### 2.1 Study design and population

This was a retrospective single-center cohort study conducted at West China Hospital of Sichuan University. The study population included patients diagnosed with sepsis or septic shock according to the Sepsis-3.0 criteria, identified through a systematic review of hospital discharge diagnoses and clinical documentation in the Hospital Information System (HIS), who were admitted to the hospital from January 2024 to December 2025.

**Inclusion criteria:** (1) Aged ≥18 years; (2) Meets the Sepsis-3.0 diagnostic criteria; (3) Hospital stay ≥7 days; (4) Complete clinical and laboratory data; (5) Approved by the Ethics Committee of West China Hospital of Sichuan University (Approval No.: 20220868).

**Exclusion criteria:** (1) Aged < 18 years; (2) Previous history of chronic kidney disease (such as glomerulonephritis, nephrotic syndrome, chronic renal failure), kidney transplantation, or pregnant women; (3) Complicated with malignant tumors, autoimmune diseases, or renal obstructive diseases; (4) Renal injury caused by non-septic factors (such as drugs, trauma, contrast agents); and (5) Incomplete clinical or laboratory data.

SA-AKI was defined according to the 2012 KDIGO guidelines: (1) Scr increased by ≥26.5 μmol/L (≥0.3 mg/dl) within 48 h; or (2) Scr increased to ≥1.5 times the baseline value within 7 days; or (3) Urine output < 0.5 ml/kg/h for 6 consecutive hours.

### 2.2 Data collection

Clinical data were extracted from the Hospital Information System (HIS) and Laboratory Information Management System (LIS), including:

1) **Basic demographic information:** Age, gender;
2) **First serum laboratory indicators after admission:** First serum test results after admission, including routine blood tests [white blood cell count (WBC), neutrophil count (NEUT), lymphocyte count (LYMPH), red blood cell distribution width (RDW), platelet count (PLT)], inflammatory markers [C-reactive protein (CRP), procalcitonin (PCT), interleukin-6 (IL-6)], renal function indicators [Scr, blood urea nitrogen (BUN), uric acid (UA)], liver function indicators [alanine aminotransferase (ALT), aspartate aminotransferase (AST), total bilirubin (TBIL)], coagulation function indicators [prothrombin time (PT), activated partial thromboplastin time (APTT), D-dimer (DD), fibrin/fibrinogen degradation products (FDP), antithrombin III (ATIII)], electrolyte indicators [potassium (K), sodium (Na), carbon dioxide combining power (CO_2_-CP)], and other indicators [lactate (LAC), albumin (Alb), myoglobin (MYO), phosphorus (PO_4_)].

### 2.3 Statistical analysis

Statistical analysis was performed using R software (version 4.2.0) and SPSS 26.0 software. Continuous variables with normal distribution were expressed as mean ± standard deviation (SD), and comparisons between groups were performed using independent sample *t*-test; continuous variables with non-normal distribution were expressed as median [interquartile range (IQR)], and comparisons between groups were performed using Mann–Whitney *U*-test. Categorical variables were expressed as frequency (percentage), and comparisons between groups were performed using chi-square test or Fisher's exact test.

1) **Data splitting:** The study population was randomly divided into a training cohort (70%) and a validation cohort (30%) using the “caret” package in R software, ensuring the consistency of SA-AKI incidence between the two cohorts.
2) **Variable screening:** To avoid overfitting and select the most robust features, Least Absolute Shrinkage and Selection Operator (LASSO) regression was applied to screen potential predictors exclusively from the first serum laboratory indicators.
3) **Model construction:** Multivariate logistic regression analysis was performed using the “rms” package to identify independent risk factors for SA-AKI, and the predictive model was constructed. Multicollinearity among the predictors in the final model was assessed using variance inflation factors (VIFs), with *VIF* < 5 considered to indicate no evidence of significant multicollinearity.
4) **Nomogram construction:** A nomogram was constructed based on the final multivariate logistic regression model to facilitate individualized bedside estimation of SA-AKI risk.
5) **Subgroup analyses:** To evaluate model robustness, subgroup analyses were performed by stratifying patients by age (>55 years vs. ≤ 55 years, based on the cohort median) and sex (male vs. female). Model discrimination was evaluated within each subgroup using ROC curve analysis.
6) **Model validation:** Discriminative ability: Evaluated using ROC curves, and AUC, sensitivity, specificity, and optimal cutoff value were calculated;

Calibration: Evaluated using calibration curves, and the Hosmer–Lemeshow test was performed (*P* > 0.05 indicates good calibration; however, the known sensitivity of this test to sample size was taken into consideration during interpretation);

Clinical utility: Evaluated using DCA, and the net benefit under different threshold probabilities was calculated to assess the clinical application value of the model.

A two-sided *P* < 0.05 was considered statistically significant.

## 3 Results

### 3.1 Baseline characteristics of the study population

A total of 1,573 sepsis patients were enrolled, including 561 (35.66%) in the SA-AKI group and 1012 (64.34%) in the non-SA-AKI group. All serum biomarkers were measured from the first blood sample collected after hospital admission. Baseline characteristics (including age and gender for descriptive purposes) are shown in Table 1. Compared with the non-SA-AKI group, the SA-AKI group had significantly higher levels of CRP, PCT, IL-6, MYO, AST, PO_4_, CK, TNT, LDH, GLU, DBIL, Na, CKMB, β-HBA, K, HBDH, TG, RDW-CV, RDW-SD, WBC, NEUT, APTT, FDP, INR, and DD (all *P* < 0.05), and significantly lower levels of CHOL, TP, IBIL, GLB, CA, ALB, CO_2_-CP, PLT, and ATIII (all *P* < 0.05). No significant differences in age, gender, ALT, ALP, GGT, MG, RBC, HGB, MCV, MCH, MCHC, MONO, BASO, FIB, or PT were observed between groups (all *P* > 0.05).

**Table 1:** Baseline characteristics of the study population stratified by sepsis-associated acute kidney injury (SA-AKI) status.

Variables	Total (*n* = 1,573)	Non-SA-AKI (*n* = 1,012)	SA-AKI (*n* = 561)	*P*-value
Demographic data				
Gender, *n* (%)				
Male	907 (57.66%)	536 (52.96%)	371 (66.13%)	< 0.001
Female	666 (42.34%)	476 (47.04%)	190 (33.87%)	
Age (years), median [IQR]	55.00 [44.00; 68.00]	53.00 [42.00; 66.00]	60.00 [48.00; 72.00]	< 0.001
Inflammatory markers				
C-reactive protein (CRP, mg/L), median [IQR]	89.80 [32.20; 161.00]	81.40 [23.58; 147.00]	104.00 [52.00; 185.00]	< 0.001
Interleukin-6 (IL-6, pg/ml), median [IQR]	67.57 [29.80; 172.10]	67.57 [25.76; 118.40]	74.10 [38.87; 326.90]	< 0.001
Procalcitonin (PCT, ng/ml), median [IQR]	3.41 [0.63; 22.50]	2.89 [0.57; 20.89]	14.46 [3.46; 47.30]	< 0.001
Biochemical indicators				
Alanine aminotransferase (ALT, U/L), median [IQR]	31.00 [17.00; 69.00]	30.00 [17.00; 61.25]	33.00 [15.00; 82.00]	0.172
Aspartate aminotransferase (AST, U/L), median [IQR]	41.00 [23.00; 87.00]	36.00 [23.00; 69.25]	51.00 [24.00; 146.00]	< 0.001
Phosphorus (PO_4_, mmol/L), median [IQR]	0.92 [0.67; 1.21]	0.86 [0.64; 1.07]	1.13 [0.76; 1.64]	< 0.001
Cholesterol (CHOL, mmol/L), median [IQR]	2.83 [2.08; 3.70]	2.96 [2.18; 3.89]	2.60 [1.97; 3.35]	< 0.001
Creatine kinase (CK, U/L), median [IQR]	74.00 [34.00; 258.00]	56.00 [30.00; 138.75]	159.00 [52.00; 603.00]	< 0.001
Troponin T (TNT, ng/L), median [IQR]	31.70 [16.00; 63.10]	29.50 [12.00; 39.02]	48.50 [30.40; 128.00]	< 0.001
Lactate dehydrogenase (LDH, U/L), median [IQR]	271.00 [195.00; 432.00]	252.50 [185.75; 371.75]	316.00 [219.00; 568.00]	< 0.001
Glucose (GLU, mmol/L), median [IQR]	7.29 [5.58; 9.62]	6.99 [5.52; 9.40]	7.78 [5.74; 10.26]	< 0.001
Direct bilirubin (DBIL, μmol/L), median [IQR]	7.70 [4.10; 16.80]	6.90 [3.80; 13.55]	9.50 [4.80; 24.70]	< 0.001
Sodium (Na, mmol/L), median [IQR]	136.80 [132.90; 141.10]	136.70 [132.80; 140.50]	137.40 [133.60; 142.00]	0.001
Creatine kinase-MB (CKMB, ng/mL), median [IQR]	2.01 [1.09; 3.88]	2.01 [0.93; 2.48]	2.83 [1.69; 7.22]	< 0.001
Total protein (TP, g/L), median [IQR]	61.40 [54.60; 69.30]	62.60 [55.90; 70.70]	59.70 [52.70; 66.20]	< 0.001
Indirect bilirubin (IBIL, μmol/L), median [IQR]	5.40 [3.30; 8.50]	5.70 [3.50; 8.62]	4.80 [2.70; 7.90]	< 0.001
Alkaline phosphatase (ALP, U/L), median [IQR]	97.00 [69.00; 158.00]	97.00 [71.00; 163.25]	96.00 [67.00; 152.00]	0.134
Beta-hydroxybutyrate (β-HBA, mmol/L), median [IQR]	0.19 [0.09; 0.48]	0.17 [0.08; 0.44]	0.24 [0.12; 0.60]	< 0.001
Gamma-glutamyl transpeptidase (GGT, U/L), median [IQR]	55.00 [26.00; 137.00]	54.50 [25.00; 135.25]	55.00 [27.00; 138.00]	0.551
Globulin (GLB, g/L), median [IQR]	29.40 [25.10; 34.30]	29.80 [25.87; 34.70]	28.30 [24.30; 33.50]	< 0.001
Calcium (CA, mmol/L), median [IQR]	2.04 [1.91; 2.18]	2.04 [1.93; 2.18]	2.01 [1.87; 2.15]	< 0.001
Albumin (ALB, g/L), median [IQR]	31.20 [27.20; 36.20]	31.85 [27.48; 37.10]	30.50 [26.70; 34.40]	< 0.001
Potassium (K, mmol/L), median [IQR]	3.85 [3.46; 4.29]	3.76 [3.41; 4.11]	4.07 [3.57; 4.66]	< 0.001
Carbon dioxide combining power (CO_2_-CP, mmol/L), median [IQR]	19.80 [16.10; 23.40]	21.30 [18.10; 24.30]	16.60 [12.40; 20.30]	< 0.001
Magnesium (MG, mmol/L), median [IQR]	0.79 [0.68; 0.89]	0.79 [0.70; 0.87]	0.79 [0.67; 0.95]	0.105
Total bilirubin (TBIL, μmol/L), median [IQR]	14.40 [8.80; 26.30]	13.70 [8.70; 23.33]	16.10 [8.90; 33.80]	0.001
Hydroxybutyrate dehydrogenase (HBDH, U/L), median [IQR]	208.00 [152.00; 332.00]	193.00 [145.00; 292.25]	241.00 [170.00; 426.00]	< 0.001
Triglyceride (TG, mmol/L), median [IQR]	1.39 [0.95; 2.11]	1.33 [0.95; 1.94]	1.49 [0.99; 2.40]	0.001
Hematological indicators				
Red blood cell (RBC, 10^12^/L), median [IQR]	3.61 [3.00; 4.21]	3.65 [3.02; 4.22]	3.55 [2.92; 4.18]	0.26
Hemoglobin (HGB, g/L), median [IQR]	106.00 [88.00; 125.00]	106.00 [89.00; 125.00]	105.00 [87.00; 126.00]	0.959
Mean corpuscular volume (MCV, fL), median [IQR]	90.25 [85.80; 94.70]	90.22 [85.70; 94.12]	90.30 [85.90; 95.50]	0.087
Mean corpuscular hemoglobin (MCH, pg), median [IQR]	29.90 [28.50; 31.40]	29.80 [28.30; 31.30]	30.20 [28.70; 31.50]	0.006
Mean corpuscular hemoglobin concentration (MCHC, g/L), median [IQR]	331.00 [320.00; 341.00]	331.00 [320.00; 340.00]	331.00 [321.00; 342.00]	0.059
Red blood cell distribution width-coefficient of variation (RDW-CV, %), median [IQR]	14.50 [13.50; 16.00]	14.40 [13.40; 15.80]	14.70 [13.70; 16.30]	< 0.001
Red blood cell distribution width-standard deviation (RDW-SD, fL), median [IQR]	46.50 [42.80; 51.40]	46.00 [42.60; 50.90]	47.40 [43.20; 51.90]	0.001
Platelet (PLT, 10^9^/L), median [IQR]	136.00 [73.00; 211.00]	147.00 [91.00; 227.00]	103.00 [51.00; 178.00]	< 0.001
White blood cell (WBC, 10^9^/L), median [IQR]	10.46 [6.64; 16.82]	9.29 [5.96; 14.92]	12.72 [8.15; 19.30]	< 0.001
Neutrophil (NEUT, 10^9^/L), median [IQR]	8.69 [4.93; 14.65]	7.56 [4.27; 12.65]	10.68 [6.63; 17.21]	< 0.001
Lymphocyte (LYMPH, 10^9^/L), median [IQR]	0.87 [0.55; 1.38]	0.92 [0.59; 1.42]	0.80 [0.50; 1.29]	0.002
Monocyte (MONO, 10^9^/L), median [IQR]	0.41 [0.24; 0.69]	0.41 [0.24; 0.65]	0.43 [0.23; 0.78]	0.108
Eosinophil (EO, 10^9^/L), median [IQR]	0.03 [0.01; 0.10]	0.03 [0.01; 0.10]	0.03 [0.00; 0.09]	0.039
Basophil (BASO, 10^9^/L), median [IQR]	0.01 [0.00; 0.02]	0.01 [0.00; 0.02]	0.01 [0.00; 0.02]	0.928
Coagulation indicators				
Fibrinogen (FIB, g/L), median [IQR]	4.07 [2.88; 5.51]	4.07 [2.88; 5.39]	4.15 [2.83; 5.62]	0.773
Antithrombin III (ATIII, %), median [IQR]	64.40 [51.50; 78.10]	65.65 [55.77; 80.60]	59.50 [46.30; 73.70]	< 0.001
Activated partial thromboplastin time (APTT, s), median [IQR]	33.60 [29.20; 41.00]	32.70 [28.60; 37.50]	37.30 [31.30; 47.20]	< 0.001
Fibrin/fibrinogen degradation products (FDP, mg/L), median [IQR]	13.80 [7.60; 25.20]	13.65 [6.40; 20.70]	17.60 [9.80; 33.40]	< 0.001
International normalized ratio (INR), median [IQR]	1.21 [1.08; 1.39]	1.18 [1.05; 1.31]	1.31 [1.14; 1.58]	< 0.001
D-dimer (DD, mg/L), median [IQR]	5.56 [2.43; 10.32]	5.00 [2.12; 8.96]	7.83 [3.50; 14.76]	< 0.001
Prothrombin time (PT, s), median [IQR]	13.90 [12.40; 15.90]	13.60 [12.20; 15.00]	14.90 [13.20; 17.80]	< 0.001

IQR, interquartile range; SA-AKI was defined according to the 2012 KDIGO criteria. Continuous variables are presented as median [IQR] and compared using the Mann–Whitney *U*-test; categorical variables are presented as *n* (%) and compared using the chi-square test.

### 3.2 Baseline characteristics of the training and validation cohorts

The study population was randomly divided into a training cohort (*n* = 1,102) and validation cohort (*n* = 471). As shown in Table 2, no significant differences in demographic data (age, gender), clinical characteristics, serum laboratory indicators, or SA-AKI incidence were observed between cohorts (all *P* > 0.05), indicating reasonable data splitting and good comparability.

**Table 2:** Baseline characteristics of the training and validation cohorts.

Variables	Total (*n* = 1,573)	Training cohort (*n* = 1,102, 70%)	Validation cohort (*n* = 471, 30%)	*P*-value
Demographic data				
Gender, *n* (%)				
Male	907 (57.66%)	652 (59.17%)	255 (54.14%)	0.073
Female	666 (42.34%)	450 (40.83%)	216 (45.86%)	
Age (years), median [IQR]	55.00 [44.00; 68.00]	55.00 [44.00; 69.00]	55.00 [44.00; 66.00]	0.492
Sepsis-associated acute kidney injury (SA-AKI), n (%)	561 (35.66%)	386 (35.02%)	175 (37.15%)	0.566
Inflammatory markers				
C-reactive protein (CRP, mg/L), median [IQR]	89.80 [32.20; 161.00]	86.30 [30.30; 163.00]	96.10 [38.25; 156.00]	0.386
Interleukin-6 (IL-6, pg/ml), median [IQR]	67.57 [29.80; 172.10]	67.57 [29.25; 163.65]	67.57 [31.19; 187.45]	0.645
Procalcitonin (PCT, ng/ml), median [IQR]	3.41 [0.63; 22.50]	3.28 [0.61; 21.95]	3.65 [0.68; 23.80]	0.417
Biochemical indicators				
Alanine aminotransferase (ALT, U/L), median [IQR]	31.00 [17.00; 69.00]	30.00 [17.00; 68.00]	31.00 [17.00; 69.50]	0.533
Aspartate aminotransferase (AST, U/L), median [IQR]	41.00 [23.00; 87.00]	41.00 [22.00; 84.75]	42.00 [24.00; 92.00]	0.143
Phosphorus (PO_4_, mmol/L), median [IQR]	0.92 [0.67; 1.21]	0.92 [0.68; 1.21]	0.91 [0.66; 1.21]	0.384
Cholesterol (CHOL, mmol/L), median [IQR]	2.83 [2.08; 3.70]	2.86 [2.13; 3.75]	2.71 [2.00; 3.60]	0.075
Creatine kinase (CK, U/L), median [IQR]	74.00 [34.00; 258.00]	73.00 [34.00; 260.00]	81.00 [36.00; 249.50]	0.183
Troponin T (TNT, ng/L), median [IQR]	31.70 [16.00; 63.10]	31.70 [16.00; 61.50]	31.70 [16.00; 65.20]	0.712
Lactate dehydrogenase (LDH, U/L), median [IQR]	271.00 [195.00; 432.00]	270.00 [195.00; 418.75]	272.00 [197.50; 462.50]	0.186
Glucose (GLU, mmol/L), median [IQR]	7.29 [5.58; 9.62]	7.31 [5.59; 9.65]	7.25 [5.56; 9.58]	0.893
Direct bilirubin (DBIL, μmol/L), median [IQR]	7.70 [4.10; 16.80]	7.60 [4.00; 16.50]	7.80 [4.20; 17.20]	0.645
Sodium (Na, mmol/L), median [IQR]	136.80 [132.90; 141.10]	136.70 [132.80; 141.00]	137.00 [133.10; 141.30]	0.623
Creatine kinase-MB (CKMB, ng/mL), median [IQR]	2.01 [1.09; 3.88]	2.01 [1.08; 3.85]	2.01 [1.10; 3.92]	0.917
Total protein (TP, g/L), median [IQR]	61.40 [54.60; 69.30]	61.50 [54.80; 69.50]	61.20 [54.20; 69.00]	0.762
Indirect bilirubin (IBIL, μmol/L), median [IQR]	5.40 [3.30; 8.50]	5.40 [3.30; 8.40]	5.40 [3.20; 8.60]	0.835
Alkaline phosphatase (ALP, U/L), median [IQR]	97.00 [69.00; 158.00]	97.00 [69.00; 157.00]	97.00 [69.00; 159.00]	0.924
Beta-hydroxybutyrate (β-HBA, mmol/L), median [IQR]	0.19 [0.09; 0.48]	0.18 [0.09; 0.47]	0.20 [0.10; 0.49]	0.516
Gamma-glutamyl transpeptidase (GGT, U/L), median [IQR]	55.00 [26.00; 137.00]	55.00 [26.00; 136.00]	55.00 [26.00; 138.00]	0.879
Globulin (GLB, g/L), median [IQR]	29.40 [25.10; 34.30]	29.50 [25.20; 34.40]	29.20 [25.00; 34.10]	0.683
Calcium (CA, mmol/L), median [IQR]	2.04 [1.91; 2.18]	2.04 [1.91; 2.18]	2.03 [1.90; 2.17]	0.735
Albumin (ALB, g/L), median [IQR]	31.20 [27.20; 36.20]	31.30 [27.30; 36.30]	31.00 [27.00; 36.00]	0.582
Potassium (K, mmol/L), median [IQR]	3.85 [3.46; 4.29]	3.84 [3.45; 4.28]	3.87 [3.48; 4.31]	0.591
Carbon dioxide combining power (CO_2_-CP, mmol/L), median [IQR]	19.80 [16.10; 23.40]	19.80 [15.93; 23.40]	19.80 [16.40; 23.35]	0.943
Magnesium (MG, mmol/L), median [IQR]	0.79 [0.68; 0.89]	0.79 [0.68; 0.88]	0.79 [0.68; 0.90]	0.814
Total bilirubin (TBIL, μmol/L), median [IQR]	14.40 [8.80; 26.30]	14.30 [8.70; 26.00]	14.60 [8.90; 26.80]	0.726
Hydroxybutyrate dehydrogenase (HBDH, U/L), median [IQR]	208.00 [152.00; 332.00]	207.00 [151.00; 330.00]	210.00 [153.00; 335.00]	0.691
Triglyceride (TG, mmol/L), median [IQR]	1.39 [0.95; 2.11]	1.40 [0.96; 2.13]	1.38 [0.94; 2.08]	0.758
Lactate (LAC, mmol/L), median [IQR]	2.40 [1.50; 4.10]	2.40 [1.50; 4.00]	2.40 [1.50; 4.20]	0.679
Hematological indicators				
Red blood cell (RBC, 10^12^/L), median [IQR]	3.61 [3.00; 4.21]	3.62 [3.01; 4.22]	3.59 [2.98; 4.18]	0.647
Hemoglobin (HGB, g/L), median [IQR]	106.00 [88.00; 125.00]	106.00 [88.00; 125.00]	105.00 [87.00; 126.00]	0.892
Mean corpuscular volume (MCV, fL), median [IQR]	90.25 [85.80; 94.70]	90.20 [85.70; 94.60]	90.30 [85.90; 94.80]	0.913
Mean corpuscular hemoglobin (MCH, pg), median [IQR]	29.90 [28.50; 31.40]	29.90 [28.50; 31.30]	29.90 [28.50; 31.50]	0.867
Mean corpuscular hemoglobin concentration (MCHC, g/L), median [IQR]	331.00 [320.00; 341.00]	331.00 [320.00; 340.00]	331.00 [321.00; 342.00]	0.759
Red blood cell distribution width-coefficient of variation (RDW-CV, %), median [IQR]	14.50 [13.50; 16.00]	14.60 [13.50; 16.00]	14.40 [13.50; 15.80]	0.317
Red blood cell distribution width-standard deviation (RDW-SD, fL), median [IQR]	46.50 [42.80; 51.40]	46.60 [42.90; 51.50]	46.30 [42.70; 51.20]	0.684
Platelet (PLT, 10^9^/L), median [IQR]	136.00 [73.00; 211.00]	141.50 [74.00; 220.00]	125.00 [73.00; 190.50]	0.025
White blood cell (WBC, 10^9^/L), median [IQR]	10.46 [6.64; 16.82]	10.46 [6.59; 16.58]	10.51 [6.77; 17.11]	0.512
Neutrophil (NEUT, 10^9^/L), median [IQR]	8.69 [4.93; 14.65]	8.69 [4.86; 14.42]	8.67 [5.04; 15.23]	0.488
Lymphocyte (LYMPH, 10^9^/L), median [IQR]	0.87 [0.55; 1.38]	0.88 [0.56; 1.39]	0.85 [0.53; 1.35]	0.419
Monocyte (MONO, 10^9^/L), median [IQR]	0.41 [0.24; 0.69]	0.41 [0.24; 0.68]	0.41 [0.24; 0.70]	0.926
Eosinophil (EO, 10^9^/L), median [IQR]	0.03 [0.01; 0.10]	0.03 [0.01; 0.10]	0.03 [0.01; 0.10]	0.875
Basophil (BASO, 10^9^/L), median [IQR]	0.01 [0.00; 0.02]	0.01 [0.00; 0.02]	0.01 [0.00; 0.02]	0.963
Coagulation indicators				
Fibrinogen (FIB, g/L), median [IQR]	4.07 [2.88; 5.51]	4.08 [2.89; 5.52]	4.05 [2.86; 5.48]	0.816
Antithrombin III (ATIII, %), median [IQR]	64.40 [51.50; 78.10]	64.40 [51.40; 77.90]	64.40 [51.90; 78.30]	0.936
Activated partial thromboplastin time (APTT, s), median [IQR]	33.60 [29.20; 41.00]	33.50 [29.10; 40.80]	33.80 [29.40; 41.50]	0.573
Fibrin/fibrinogen degradation products (FDP, mg/L), median [IQR]	13.80 [7.60; 25.20]	13.80 [7.50; 24.17]	13.80 [7.75; 27.05]	0.122
International normalized ratio (INR), median [IQR]	1.21 [1.08; 1.39]	1.21 [1.08; 1.38]	1.22 [1.09; 1.41]	0.562
D-dimer (DD, mg/L), median [IQR]	5.56 [2.43; 10.32]	5.56 [2.45; 9.96]	5.56 [2.40; 11.57]	0.245
Prothrombin time (PT, s), median [IQR]	13.90 [12.40; 15.90]	13.90 [12.40; 15.70]	13.90 [12.50; 16.30]	0.189

IQR, interquartile range; SA-AKI, sepsis-associated acute kidney injury. The study population was randomly divided into the training cohort (70%) and validation cohort (30%). Continuous variables are presented as median (IQR) and compared using the Mann–Whitney *U*-test; categorical variables are presented as *n* (%) and compared using the chi-square test. All *P* values >0.05 indicate no significant differences between the two cohorts.

### 3.3 LASSO regression for variable screening

**Figure 2:**
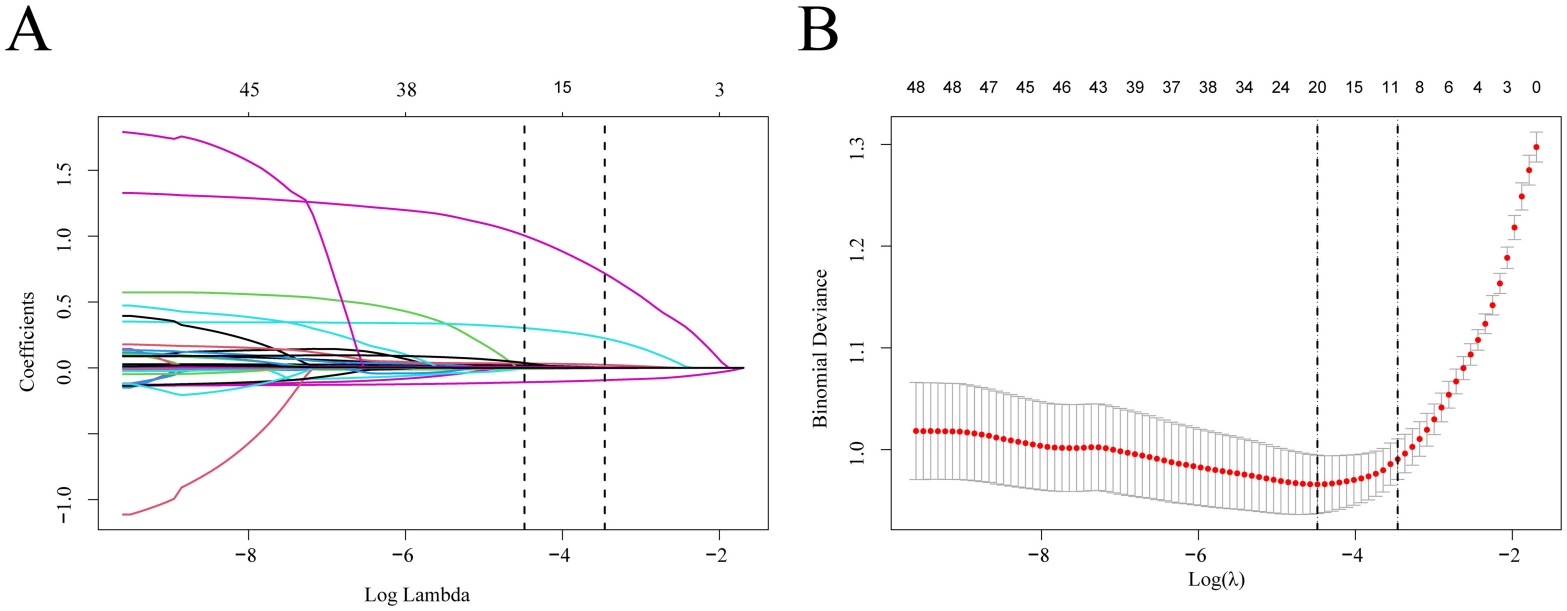
LASSO regression for variable screening. **(A)** Coefficient profile plot of LASSO regression: each curve represents the change in coefficient of a candidate serum variable with increasing log(lambda) value; **(B)** ten-fold cross-validation plot for determining the optimal lambda value: the vertical dashed line indicates lambda.min (0.01129457), corresponding to the minimum cross-validation error, and 10 serum variables with non-zero coefficients were selected as potential predictive factors for SA-AKI.

A total of 56 initial serum laboratory indicators were included in LASSO regression. The coefficient profile plot showed regression coefficients gradually decreased with increasing lambda value (Figure 2A). Ten-fold cross-validation determined the optimal lambda value (lambda.min = 0.01129457), and 10 serum indicators with non-zero coefficients were selected as potential predictive factors for SA-AKI: MYO, ALT, PO_4_, Na, TP, K, CO_2_, PLT, NEUT, and DD (Figure 2B).

### 3.4 Multivariate logistic regression analysis of independent risk factors

The 10 serum indicators screened by LASSO regression were included in multivariate logistic regression. After stepwise elimination, 8 serum indicators were retained in the final regression equation (Table 3): MYO (adjusted *OR* = 1.001, 95% *CI*: 1.000–1.001, *P* < 0.001), ALT (adjusted *OR* = 1.001, 95% *CI*: 1.000–1.001, *P* = 0.025), PO_4_(adjusted *OR* = 3.246, 95% *CI*: 2.273–4.635, *P* < 0.001), Na (adjusted *OR* = 1.029, 95% *CI*: 1.005–1.054, *P* = 0.019), K (adjusted *OR* = 1.434, 95% *CI*: 1.140–1.802, *P* = 0.002), CO_2_-CP (adjusted *OR* = 0.880, 95% *CI*: 0.852–0.909, *P* < 0.001), PLT (adjusted *OR* = 0.996, 95% *CI*: 0.995–0.998, *P* < 0.001), and NEUT (adjusted *OR* = 1.050, 95% *CI*: 1.029–1.072, *P* < 0.001). Age and gender were not included in the regression model at any stage. Multicollinearity assessment using variance inflation factors (VIFs) showed no evidence of significant collinearity among the 8 predictors (all *VIF* < 1.2, range: 1.020–1.182; Supplementary Table 1), supporting the stability of the regression coefficients.

**Table 3:** Multivariate logistic regression analysis of independent risk factors for sepsis-associated acute kidney injury (SA-AKI).

Variables	β	*SE*	*Z*-value	*P*-value	Adjusted *OR*	95% ***CI***	
						Lower	Upper
Myoglobin (MYO, ng/ml)	0.001	0.00012	5.933	< 0.001	1.001	1	1.001
Alanine aminotransferase (ALT, U/L)	0.001	0.00035	2.237	0.025	1.001	1	1.001
Phosphorus (PO_4_, mmol/L)	1.177	0.18172	6.479	< 0.001	3.246	2.273	4.635
Sodium (Na, mmol/L)	0.029	0.01233	2.342	0.019	1.029	1.005	1.054
Potassium (K, mmol/L)	0.36	0.11674	3.085	0.002	1.434	1.14	1.802
Carbon dioxide combining power (CO_2_-CP, mmol/L)	−0.128	0.01667	−7.677	< 0.001	0.88	0.852	0.909
Platelet (PLT, 10^9^/L)	−0.004	0.00079	−4.782	< 0.001	0.996	0.995	0.998
Neutrophil (NEUT, 10^9^/L)	0.049	0.01034	4.724	< 0.001	1.05	1.029	1.072

This table presents the results of multivariate logistic regression analysis. A total of 10 variables screened by LASSO regression were included in the initial model, and 8 variables were finally retained in the regression equation after stepwise elimination (*P* < 0.05). β, regression coefficient; SE, standard error; OR, odds ratio; CI, confidence interval. Variables are consistent with those in Tables 1, 2, with abbreviations defined as follows: MYO, myoglobin; ALT, alanine aminotransferase; PO_4_, phosphorus; Na, sodium; K, potassium; CO_2_-CP, carbon dioxide combining power; PLT, platelet; NEUT, neutrophil. Statistical significance was set at two-tailed *P* < 0.05.

### 3.5 Model performance evaluation

#### 3.5.1 Model discriminative ability

**Figure 3:**
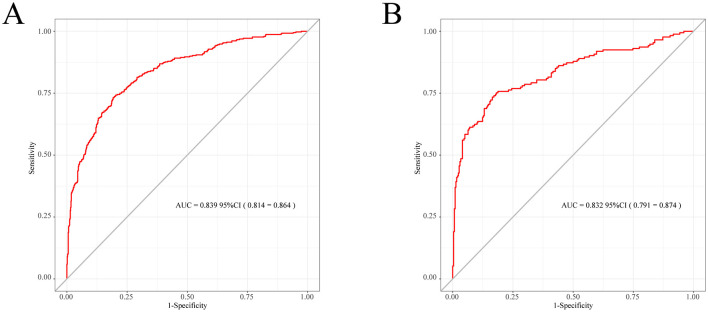
Receiver operating characteristic (ROC) curves of the predictive model. **(A)** ROC curve for the Training Cohort (*AUC* = 0.839, 95% *CI*: 0.814–0.864). **(B)** ROC curve for the validation cohort (*AUC* = 0.832, 95% *CI*: 0.791–0.874). The red line represents the performance of the model. AUC, area under the ROC curve; CI, confidence interval.

ROC curve analysis showed the model's AUC was 0.839 (95% *CI*: 0.814–0.864) in the training cohort, with a sensitivity of 78.6% and specificity of 79.2% at the optimal cutoff value of 0.45 (Figure 3A). In the validation cohort, the AUC was 0.832 (95% *CI*: 0.791–0.874), with a sensitivity of 77.1% and specificity of 78.5% (Figure 3B), indicating good discriminative ability in both cohorts.

#### 3.5.2 Model calibration

**Figure 4:**
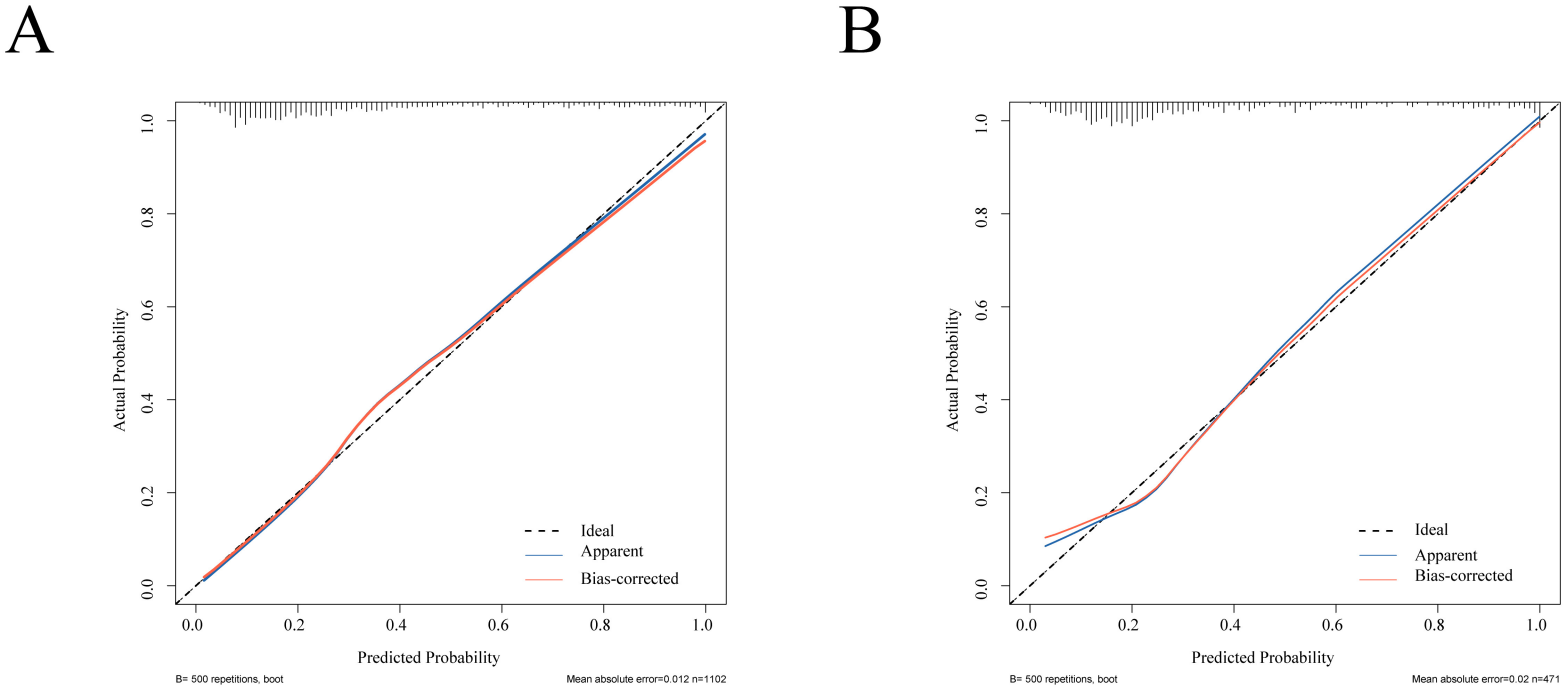
Calibration curves of the predictive model. **(A)** Calibration curve for the training cohort. **(B)** Calibration curve for the validation cohort. The *x*-axis represents the predicted probability of SA-AKI, and the *y*-axis represents the actual observed probability. The diagonal dashed line represents a perfect prediction by an ideal model. The solid blue line represents the apparent performance, and the red line represents the bias-corrected performance.

Calibration curves showed acceptable graphical agreement between predicted SA-AKI probabilities and actual observations in both the training and validation cohorts (Figures 4A, B). The bias-corrected calibration curves closely approximated the ideal diagonal line, indicating reasonable consistency between the predicted and actual risks of SA-AKI. Hosmer–Lemeshow goodness-of-fit test results were *P* = 0.1056 (training cohort, indicating good calibration) and *P* = 0.002398 (validation cohort). Although the Hosmer–Lemeshow test was statistically significant in the validation cohort, the calibration curve demonstrated acceptable visual agreement between predicted and observed probabilities, and decision curve analysis (DCA) confirmed clinical net benefit. The significant Hosmer–Lemeshow result likely reflects the known sensitivity of this test to sample size and the number of groups used for stratification (8); external validation in larger cohorts is needed to further assess calibration stability.

#### 3.5.3 Clinical utility of the model

**Figure 5:**
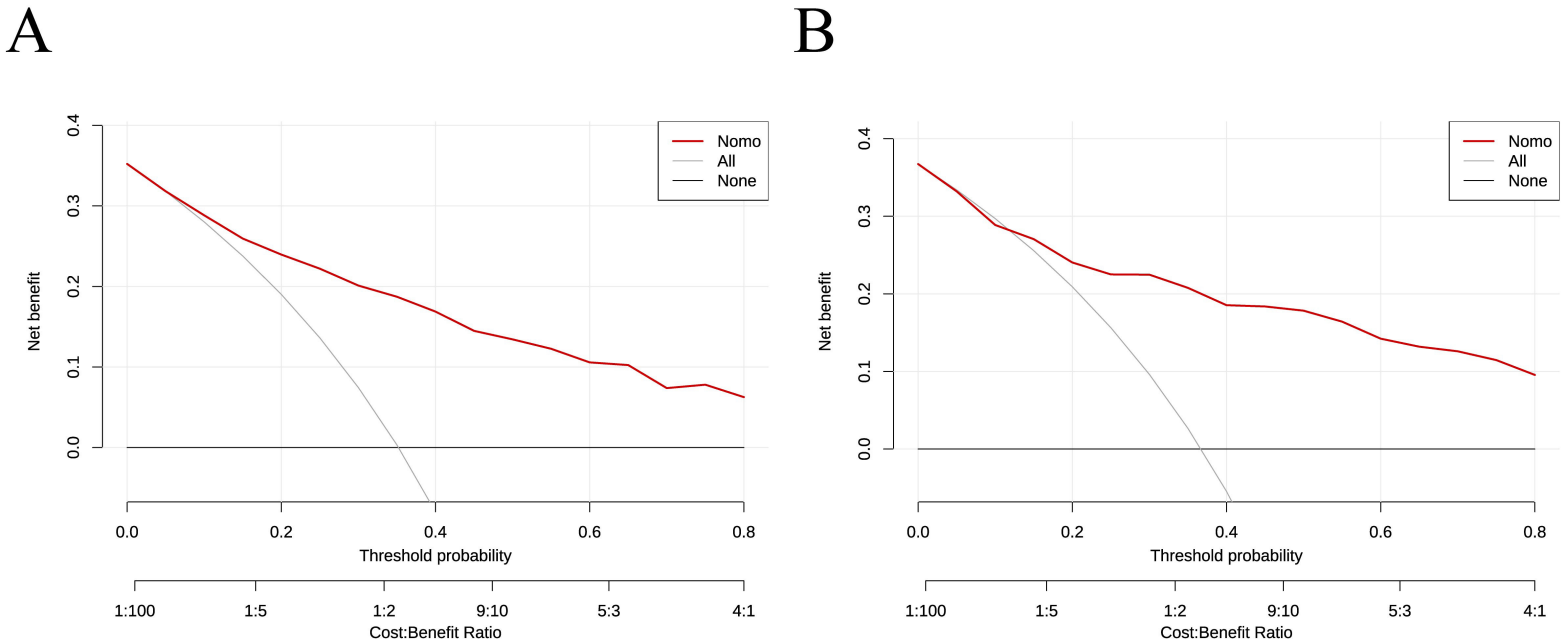
Decision curve analysis (DCA) for the predictive model. **(A)** DCA for the training cohort. **(B)** DCA for the validation cohort. The *y*-axis measures the net benefit. The red line represents the predictive model. The gray line represents the assumption that all patients have SA-AKI (“Treat All”), and the black horizontal line represents the assumption that no patients have SA-AKI (“Treat None”). The model shows a higher net benefit across the threshold probability range of approximately 5% to 80%.

DCA showed that within a threshold probability range of 5–80%, the model's net benefit was significantly higher than that of “treat all” or “treat none” strategies in both the training and validation cohorts (Figures 5A, B), indicating substantial clinical utility and potential benefits for patient management.

#### 3.5.4 Nomogram for individualized risk prediction

**Figure 6:**
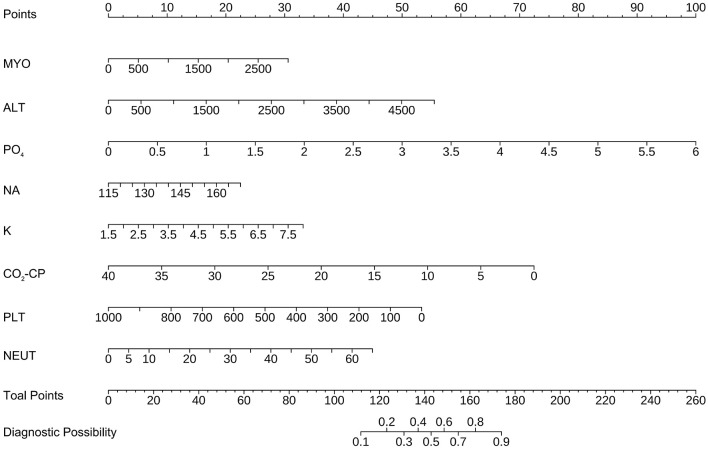
Nomogram for predicting the probability of sepsis-associated acute kidney injury (SA-AKI). To estimate an individual patient's risk, locate each biomarker value on its corresponding axis and draw a vertical line upward to the “Points” scale to obtain the assigned score. After summing the scores of all eight variables, locate the total score on the “Total Points” axis and draw a vertical line downward to the “Diagnostic Possibility” axis to read the predicted probability of SA-AKI. **Abbreviations**: MYO, myoglobin; ALT, alanine aminotransferase; PO_4_, phosphorus; Na, sodium; K, potassium; CO_2_-CP, carbon dioxide combining power; PLT, platelet count; NEUT, neutrophil count.

Based on the final multivariate logistic regression model, a nomogram was constructed to facilitate individualized prediction of SA-AKI risk (Figure 6). Each of the eight predictors was assigned a weighted score according to its regression coefficient, and the total score corresponded to the estimated probability of SA-AKI. Detailed usage instructions are provided in the figure legend.

#### 3.5.5 Subgroup analyses

Subgroup analyses were performed to evaluate model discrimination across age and sex strata (Supplementary Figure 1). The model maintained favorable discriminative ability in patients aged >55 years (*AUC* = 0.821, 95% *CI*: 0.790–0.852; Supplementary Figure 1A), patients aged ≤ 55 years (*AUC* = 0.865, 95% *CI*: 0.836–0.894; Supplementary Figure 1B), males (*AUC* = 0.853, 95% *CI*: 0.828–0.879; Supplementary Figure 1C), and females (*AUC* = 0.816, 95% *CI*: 0.777–0.854; Supplementary Figure 1D). No significant interaction effects were observed across the four subgroups, supporting the internal stability of the model across these clinically relevant demographic strata.

## 4 Discussion

In this retrospective cohort study, we developed and internally validated a serum-biomarker–exclusive prediction model for SA-AKI in sepsis patients. Using eight routine first serum laboratory indicators after hospital admission (MYO, ALT, PO_4_, Na, K, CO_2_-CP, PLT, NEUT), the model achieved favorable discrimination (AUC 0.839 in the training cohort; 0.832 in the validation cohort), acceptable graphical calibration, and meaningful clinical utility across a broad range of decision thresholds. A nomogram was constructed to facilitate individualized bedside risk estimation, and subgroup analyses showed stable discriminative performance across age and sex strata.

### 4.1 Biological plausibility of predictors

The selected variables collectively capture the multifactorial pathophysiology of SA-AKI:

**Metabolic and electrolyte derangements:** Hyperphosphatemia (PO_4_), the strongest predictor (*OR* = 3.246), precedes Scr elevation by reflecting reduced glomerular filtration and cell lysis-induced phosphate release, while directly exacerbating renal injury via calcium-phosphate precipitation and endothelial dysfunction (9). This finding is consistent with recent evidence identifying phosphorus as a robust predictor of SA-AKI (10). Electrolyte disturbances (Na, K) and acid-base imbalance (CO_2_-CP) indicate impaired renal homeostatic capacity—metabolic acidosis (low CO_2_-CP) reduces renal perfusion and promotes tubular apoptosis, aligning with its protective association (11).

**Tissue injury and hepatorenal crosstalk:** Elevated MYO may reflect rhabdomyolysis or diffuse tissue injury. Myoglobin may contribute to renal injury through multiple pathophysiological mechanisms: (1) it forms pigment casts in the renal tubules, causing mechanical obstruction and direct tubular toxicity; (2) the free iron released from myoglobin induces oxidative stress and lipid peroxidation, damaging tubular epithelial cells and renal microvascular endothelial cells (12); and (3) myoglobin promotes renal vasoconstriction by reducing nitric oxide bioavailability, leading to renal hypoperfusion. These mechanisms are amplified in the pro-inflammatory milieu of sepsis, and elevated MYO may additionally serve as a surrogate marker of systemic tissue injury severity (13). ALT elevation signals hepatic dysfunction, which amplifies systemic inflammation and microthrombosis via impaired toxin clearance, underscoring hepatorenal crosstalk in SA-AKI (12).

**Immunothrombosis:** Neutrophilia (NEUT) and thrombocytopenia (PLT) mirror sepsis-induced coagulopathy—activated neutrophils release reactive oxygen species (ROS) and neutrophil extracellular traps (NETs) to damage renal microvasculature, while platelet consumption exacerbates microthrombosis and ischemic injury (14). This coagulation-related pathway is consistent with the findings of Xie et al. (15), who identified antithrombin III—a key endogenous anticoagulant—as an independent predictor of SA-AKI, further supporting the role of immunothrombosis in SA-AKI pathogenesis (15).

### 4.2 Comparison with existing SA-AKI prediction models

The principal strength of our model lies in its exclusive reliance on first routine serum laboratory indicators after hospital admission, a feature that distinguishes it from previously published tools (Table 4).

**Table 4:** Comparison of the proposed prediction model with existing SA-AKI predictive tools.

Study	Year	Population	Key variables	Validation AUC	Core limitations
Xie et al. (15)	2021	Sepsis patients, single-center prospective	ATIII, sex, cardiovascular disease	0.986^*^	Single-center; small sample; no separate validation cohort; single-marker approach
Xin et al. (16)	2022	1,051 sepsis patients	PLT, PCT, PTA	0.887	Requires PTA (not universally measured); focused narrowly on inflammatory/coagulation markers
Zhao et al. (18)	2024	268 septic patients	NGAL, PLR, vasopressor use	0.826	Requires NGAL (non-routine biomarker); small sample size; includes clinical variables
Lin et al. (20)	2024	391 emergency sepsis patients	Vasopressor use, age, PLT, PCT, D-dimer	0.832	Emergency department-specific population; includes age (non-modifiable demographic factor)
Ge et al. (17)	2025	28,819 (multicenter, international)	UO, MV, SOFA, SCr, GCS, nephrotoxic drugs	0.839 (48 h)	Predicts moderate-to-severe SA-AKI; requires complex dual-timepoint data; machine learning model limits bedside applicability
Jiang et al. (10)	2025	46,097 (multicenter, international)	AKI stage, ΔCr, UO, furosemide, BMI, SOFA, KRT, MV, lactate, BUN, PT, age	0.852 (prospective)	Predicts persistent SA-AKI (≥48 h), not incident SA-AKI; requires 24 h clinical data; complex GBM model
Our study	2026	1,573 sepsis patients, single-center retrospective	8 routine serum biomarkers (MYO, ALT, PO_4_, Na, K, CO_2_-CP, PLT, NEUT)	0.832 (internal)	Single-center; internal validation only; lacks external validation

^*^The AUC reported by Xie et al. was derived from the training set without separate validation cohort evaluation. ATIII, antithrombin III; PLT, platelet count; PCT, procalcitonin; PTA, prothrombin time activity; NGAL, neutrophil gelatinase-associated lipocalin; PLR, platelet-to-lymphocyte ratio; UO, urine output; MV, mechanical ventilation; SOFA, Sequential Organ Failure Assessment; SCr, serum creatinine; GCS, Glasgow Coma Scale; ΔCr, 24-h creatinine change; BMI, body mass index; KRT, kidney replacement therapy; BUN, blood urea nitrogen; PT, prothrombin time; GBM, gradient boosting machine; MYO, myoglobin; ALT, alanine aminotransferase; PO_4_, phosphorus; Na, sodium; K, potassium; CO_2_-CP, carbon dioxide combining power; NEUT, neutrophil count.

Xin et al. developed a prediction model incorporating platelet count, procalcitonin, and prothrombin time activity (PTA) in 1,051 patients with sepsis, achieving excellent discrimination (*AUC* = 0.887) (16). Despite its high performance, their model requires PTA—a coagulation parameter not universally measured—and focuses narrowly on inflammatory and coagulation pathways. In contrast, our model captures a broader spectrum of pathophysiological processes, including electrolyte disturbances, tissue injury, and acid–base derangements, using only widely available laboratory tests.

Ge et al. (17) recently proposed a dual-timepoint machine learning model for moderate-to-severe SA-AKI, derived from an international multicenter cohort of 28,819 patients, with a 48-h AUC of 0.839. Although their approach benefits from large-scale data and algorithmic sophistication, its complexity—requiring 48-h and 7-day timepoints, eight machine-learning algorithms, and numerous clinical variables—may hinder straightforward implementation at the bedside. Our single-timepoint model, based solely on first serum biomarkers after admission, offers greater simplicity while showing comparable discriminative performance (validation *AUC* = 0.832).

Zhao et al. (18) constructed a nomogram incorporating neutrophil gelatinase-associated lipocalin (NGAL), platelet-to-lymphocyte ratio, and vasopressor use, achieving an AUC of 0.826. While NGAL has been advocated as an early AKI marker, its measurement is not part of routine laboratory panels in many institutions, thereby limiting the generalizability and cost-effectiveness of their tool (19). Our model circumvents the need for specialized assays.

Lin et al. developed a prediction model tailored to emergency department sepsis patients, using vasopressor use, age, platelet count, procalcitonin, and D-dimer, with a validation AUC of 0.832 (20). Although their model demonstrates comparable discrimination, its inclusion of age—a non-modifiable demographic factor—and its derivation from an emergency cohort may restrict its applicability to intensive care unit populations. By relying exclusively on serum biomarkers that reflect acute physiological perturbations, our model avoids reliance on static demographic characteristics and is directly applicable to ICU patients upon the availability of their first laboratory results.

Xie et al. conducted a prospective single-center study and established a prediction model for SA-AKI based on antithrombin III (ATIII), identifying ATIII reduction as an independent risk factor for both SA-AKI and mortality (15). This study provides valuable prospective evidence linking coagulation dysfunction to SA-AKI risk. In our own dataset, ATIII was significantly lower in the SA-AKI group (Table 1); however, it was not retained as an independent predictor in the final multivariable model after LASSO screening and stepwise elimination. This suggests that the predictive information carried by ATIII may have been partially captured by other retained variables—particularly PLT and NEUT—which also reflect sepsis-induced coagulopathy and immunothrombosis. Our model, which integrates eight biomarkers across metabolic, tissue injury, and immunothrombotic pathways, offers broader mechanistic coverage compared with a single-marker approach.

Jiang et al. (10) recently developed an interpretable gradient boosting machine model for predicting persistent SA-AKI—AKI lasting beyond 48 h—in a large multicenter cohort of 46,097 sepsis patients. Their model, which incorporates 12 features including AKI stage, SOFA score, urine output, 24-h creatinine change, and mechanical ventilation, achieved strong discriminative performance (prospective *AUC* = 0.852) and was externally validated across multiple cohorts. However, two important distinctions from our work should be noted. First, their model targets a distinct clinical endpoint—persistent rather than incident SA-AKI. Second, their model requires clinical data accumulated over the first 24 h of ICU admission, rendering it less applicable at the time of the first blood draw, which is the specific clinical niche addressed by our model. Furthermore, the inclusion of SOFA score in their final model supports the relevance of organ dysfunction assessment in SA-AKI prediction, consistent with the discussion we provide below (10).

More broadly, recent machine learning–based prediction models for SA-AKI have reported AUCs ranging from 0.77 to 0.84, yet they typically require longitudinal data streams or high-dimensional electronic health record extraction, which are not universally accessible (7, 21). Collectively, these comparisons highlight that while diverse predictive strategies exist, our model achieves comparable discriminative performance through a parsimonious set of routine first biomarkers, addressing key barriers related to data availability, complexity, and generalizability.

### 4.3 Comparison with established severity scores

We acknowledge that formal comparison with established severity scores such as SOFA and APACHE II was not a primary objective of this study. Our model was specifically designed to be deployed immediately upon the first blood draw—typically within 1–2 h of admission—well before comprehensive clinical scoring systems can be fully calculated, as these require multi-organ assessment that is usually finalized 4–6 h post-admission. The intended clinical niche of our model is precisely this early time window, complementing rather than competing with established severity scores.

Furthermore, published literature has demonstrated that the SOFA score has limited discriminative ability for SA-AKI specifically, with reported AUCs typically ranging from 0.65 to 0.75, in part because its renal sub-score is derived from the very parameters used to define AKI—serum creatinine and urine output—introducing a degree of circularity for the purpose of AKI prediction (7, 18). This inherent limitation of SOFA for SA-AKI prediction further supports the rationale for developing alternative, biomarker-based approaches.

Regarding APACHE II, this score was designed for mortality prediction and incorporates several physiological variables that were not part of our serum-biomarker-focused data extraction framework. We have acknowledged this as a limitation and recommend that future prospective studies include head-to-head comparisons with both scoring systems.

### 4.4 Clinical implications

The model's core strength lies in its feasibility: all predictors are part of standard first laboratory panels after admission, enabling timely risk stratification at the earliest available laboratory assessment. The nomogram (Figure 6) further enhances bedside applicability by allowing clinicians to estimate individual patient risk through a simple point-based scoring system without requiring electronic calculation. Clinicians can identify high-risk patients (e.g., elevated PO_4_/NEUT/MYO + reduced CO_2_-CP/PLT) to prioritize kidney-protective interventions—optimized fluid resuscitation, nephrotoxin avoidance, and electrolyte/acid-base correction—consistent with the model's demonstrated net benefit across 5%−80% threshold probabilities. Furthermore, the exclusive reliance on biomarkers that are universally part of standard admission panels means that the model can be integrated into existing Laboratory Information Systems to automatically flag high-risk patients upon the release of the first blood test results, requiring no additional data entry and thus minimizing barriers to clinical adoption.

### 4.5 Calibration considerations

The Hosmer–Lemeshow test was not significant in the training cohort (*P* = 0.1056) but reached statistical significance in the validation cohort (*P* = 0.002398). It should be noted that the Hosmer–Lemeshow test is highly sensitive to sample size and to the number of groups used for stratification (8). In cohorts of moderate size, even relatively minor deviations between observed and predicted probabilities can yield statistically significant *P*-values. The calibration curve for the validation cohort demonstrated acceptable visual agreement between predicted and actual probabilities, and the decision curve analysis confirmed consistent clinical net benefit across the entire range of clinically relevant threshold probabilities. We therefore consider the model adequately calibrated for risk stratification purposes while acknowledging that calibration stability should be further assessed in larger, independent external cohorts, with recalibration performed if necessary.

### 4.6 Subgroup robustness

The subgroup analyses demonstrated that the model maintained stable discriminative performance across age strata (>55 years: *AUC* = 0.821; ≤ 55 years: *AUC* = 0.865) and sex strata (male: *AUC* = 0.853; female: *AUC* = 0.816), supporting the internal stability of the model. All AUC values exceeded 0.81, and no significant interaction effects were observed across the four subgroups. However, due to inconsistent and non-standardized documentation of primary infection sources in the retrospective electronic medical records, we were unable to perform reliable subgroup analyses stratified by infection etiology. We recommend that future prospective studies with standardized infection source documentation evaluate model performance across different infection types (e.g., pulmonary, abdominal, urinary, bloodstream).

### 4.7 Limitations

This study has several limitations that should be carefully considered when interpreting the findings:

1) **Single-center retrospective design:** As a single-center study conducted at a large tertiary teaching hospital in western China, our cohort may over represent patients with more severe sepsis due to referral patterns, and the patient case-mix, laboratory workflows, and clinical practices may differ from those of community hospitals or healthcare settings in other geographic regions. These factors may limit generalizability. Multi-center external validation in diverse cohorts is the foremost priority for future work.
2) **Lack of external validation:** The current model has undergone random-split internal validation only. External validation in independent, multi-center, and ideally prospective cohorts is essential to confirm the model's generalizability before broader clinical implementation. Accordingly, the model should currently be considered as internally validated and requiring confirmation in independent cohorts.
3) **Unmeasured confounding factors:** As a retrospective study, several potentially important clinical variables—including detailed sepsis etiology, body mass index, detailed comorbidity profiles, concurrent medications, fluid balance, and vasopressor use—were not available for inclusion. This may result in residual confounding and limit causal interpretability. However, it should be noted that the deliberate “biomarker-only” design was a core feature of this study, intended to enable automatic, immediate, and objective risk stratification without requiring manual clinical data entry. This trade-off between comprehensiveness and bedside simplicity was a conscious design choice. Future prospective studies should evaluate whether incorporating these clinical variables can incrementally improve predictive performance.
4) **Reliance on baseline biomarkers only:** The model uses only the first set of serum laboratory indicators after admission and does not incorporate dynamic changes over the subsequent 24–48 h, which may provide additional prognostic information.
5) **Lack of comparison with established severity scores:** Direct head-to-head comparison with SOFA and APACHE II scores was not performed, as the present study focused on routinely available serum biomarkers. The retrospective reconstruction of these scores requires multiple clinical variables that were beyond the scope of our laboratory-based data extraction framework. Future prospective studies should include formal comparative analyses with both scoring systems.
6) **Inability to stratify by infection source:** Due to inconsistent and non-standardized documentation of primary infection sites in the retrospective electronic medical records, we were unable to evaluate model performance across different infection etiologies. This should be addressed in future prospective studies with systematic infection source documentation.
7) **CKD exclusion:** The exclusion of patients with pre-existing chronic kidney disease was intentional, as these patients have altered baseline biomarker profiles that may confound SA-AKI prediction. However, this limits the applicability of the model to this high-risk population. Future studies should investigate model recalibration or extension for CKD patients, potentially incorporating baseline renal function parameters such as estimated glomerular filtration rate and CKD stage.
8) **Absence of standardized lead-time data:** The exact interval between the first blood draw and KDIGO-defined AKI onset could not be consistently determined for all patients in this retrospective study. Previous literature indicates a median time of 24–48 h from admission to AKI diagnosis (22). Future prospective studies should systematically record this interval to more precisely quantify the early predictive window.
9) **Absence of granular treatment data:** The lack of detailed treatment data (e.g., fluid resuscitation volume, nephrotoxic drug exposure, timing of interventions) limits the ability to assess whether model-guided risk stratification can improve clinical outcomes.

### 4.8 Future directions

Future work should prioritize: (a) multi-center, prospective external validation in diverse geographic and healthcare settings; (b) prospective evaluation of whether incorporating dynamic biomarker trends (e.g., 24-h and 48-h changes) can improve predictive accuracy over the baseline model; (c) investigation of whether the addition of clinical variables and treatment data incrementally enhances model performance, while quantifying the trade-off between model complexity and bedside feasibility; (d) model recalibration or extension specifically for CKD populations; (e) integration of novel biomarkers (e.g., KIM-1, NGAL, CCL14) into the model framework; (f) head-to-head comparison with SOFA and APACHE II scores in prospective cohorts; and (g) interventional studies to determine whether model-guided early risk stratification translates into improved clinical outcomes through timely nephroprotective interventions.

## 5 Conclusion

We developed and internally validated a clinically applicable prediction model for SA-AKI using eight routine serum biomarkers obtained from the first laboratory assessment after hospital admission. The model exhibits favorable discriminative ability (validation *AUC* = 0.832) and clinical utility, with a nomogram provided for individualized bedside risk estimation. Subgroup analyses showed stable performance across age and sex strata. However, given the single-center retrospective design and the absence of external validation, the model should currently be considered as internally validated and requiring confirmation in independent cohorts. External validation in multi-center, diverse populations is warranted before broader clinical implementation. If externally validated, this pragmatic, biomarker-only tool may support early risk stratification and facilitate timely, targeted interventions to mitigate renal injury in sepsis patients.

## Data Availability

The original contributions presented in the study are included in the article/Supplementary material, further inquiries can be directed to the corresponding author.
