# Supplementary material for: Development and internal validation of a prediction model for early identification of sepsis-associated acute kidney injury based on admission serum biomarkers: a retrospective cohort study
Source: Front Med (Lausanne). 2026 Jun 22;13:1820595. doi: 10.3389/fmed.2026.1820595 (PMC13333455; doi:10.3389/fmed.2026.1820595)
Supplement: Supplementary file 1 [file Supplementary_file_1.docx]

**Supplementary Figure 1. Receiver operating characteristic (ROC) curves of the prediction model stratified by age and sex**


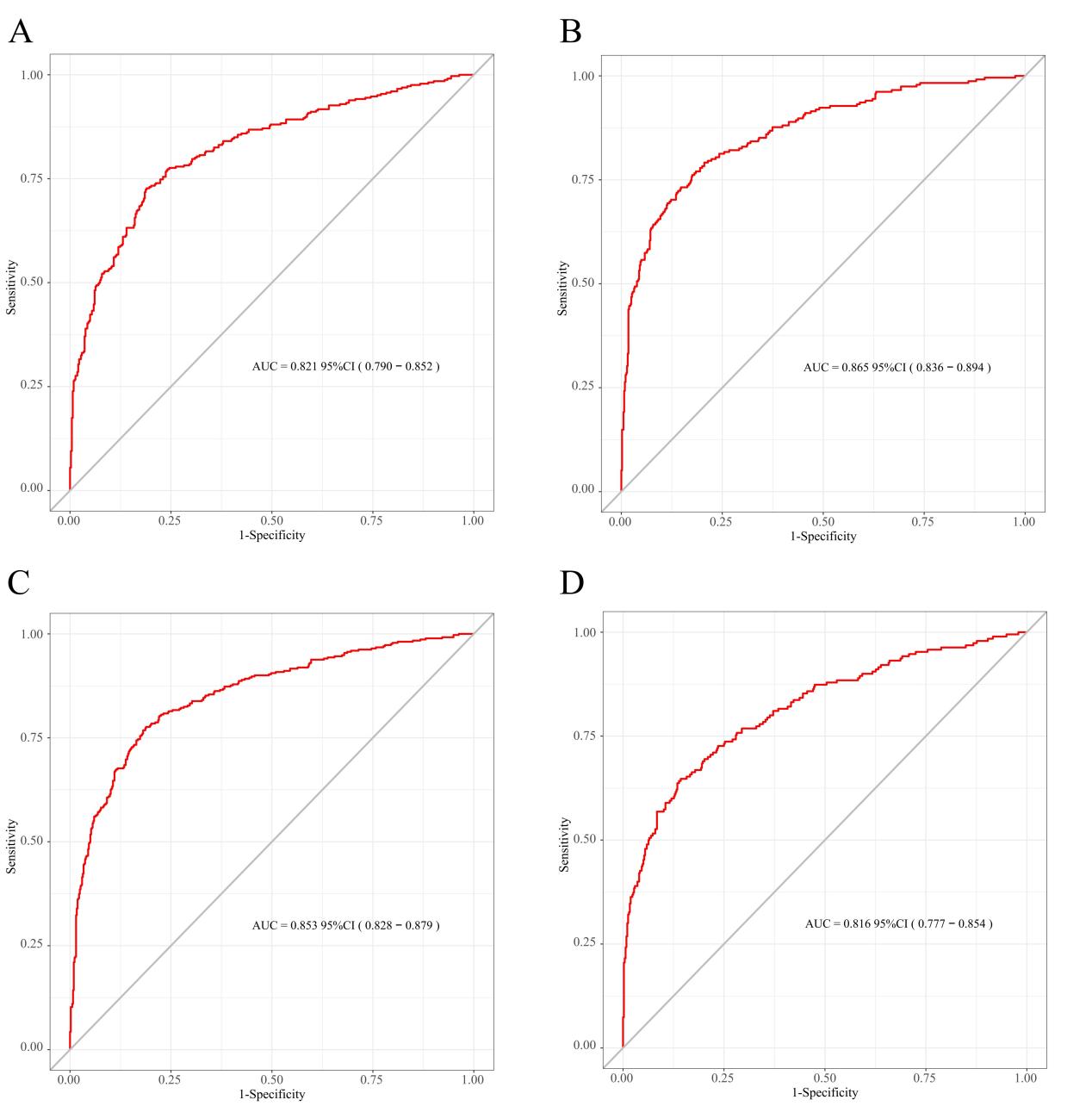


(A) Patients aged > 55 years (AUC = 0.821, 95% CI: 0.790–0.852). (B) Patients aged ≤ 55 years (AUC = 0.865, 95% CI: 0.836–0.894). (C) Male patients (AUC = 0.853, 95% CI: 0.828–0.879). (D) Female patients (AUC = 0.816, 95% CI: 0.777–0.854). The model maintained favorable discriminative ability across all four subgroups, with no significant interaction effects observed.
