# Supplementary material for: Development and internal validation of a prediction model for early identification of sepsis-associated acute kidney injury based on admission serum biomarkers: a retrospective cohort study
Source: Front Med (Lausanne). 2026 Jun 22;13:1820595. doi: 10.3389/fmed.2026.1820595 (PMC13333455; doi:10.3389/fmed.2026.1820595)
Supplement: Supplementary file 2 [file Table_1.docx]

**Supplementary Table 1. Variance Inflation Factor (VIF) values of the 8 independent predictors in the final logistic regression model**

| **Variable** | **Variance Inflation Factor (VIF)** |
| --- | --- |
| Myoglobin (MYO) | 1.05 |
| Alanine aminotransferase (ALT) | 1.02 |
| Sodium (Na) | 1.139 |
| Potassium (K) | 1.095 |
| Carbon dioxide combining power (CO₂-CP) | 1.15 |
| Phosphorus (PO₄) | 1.165 |
| Platelet (PLT) | 1.182 |
| Neutrophil (NEUT) | 1.076 |

Note: Variance inflation factor (VIF) was used to assess multicollinearity among the predictors included in the final model. A VIF value <5 was considered to indicate no evidence of significant multicollinearity. All VIF values were below 1.2, suggesting no evidence of significant multicollinearity among the final predictors.
